# Correcting misperceptions of the material benefits associated with union membership increases Americans’ interest in joining unions

**DOI:** 10.1073/pnas.2321025121

**Published:** 2024-04-29

**Authors:** Jonne Kamphorst, Robb Willer

**Affiliations:** ^a^Department of Political and Social Sciences, European University Institute, Florence 50014, Italy; ^b^Department of Sociology, Stanford University, Stanford, CA 94305

**Keywords:** unions, misperceptions, public opinion, collective action

## Abstract

How accurate are Americans’ perceptions of the material benefits associated with union membership, and do these perceptions influence their support for, and interest in joining, unions? We explore these questions in a preregistered, survey experiment conducted on a national sample, representative of the US population on a number of demographic benchmarks (*n* = 1,430). We find that Americans exhibit large and consistent underestimates of the benefits associated with unionization, as compared to evidence from the Bureau of Labor Statistics and peer-reviewed academic research. For example, 89% of Americans underestimated the life-time income premium associated with union membership, 72% underestimated the percentage of union members who receive health insurance from their employer, and 97% overestimated the average union dues rate. We next randomly assigned half of the participants to receive a brief, informational correction conveying results of academic and government research on the material benefits associated with union membership, or not. Those who received the correction reported 11.6% greater interest in joining a union, 7.8% greater support for unions, and 6.9% greater interest in helping to organize a union in their workplace, as compared to the control group. These results suggest that, overall, Americans tend to underestimate the material benefits associated with unionization, misperceptions of these benefits are causally linked to Americans’ support for unionization, and correcting these misperceptions increases a range of pro-union sentiments in the American mass public.

Studies show popular support for, and media coverage of, labor unions has grown in the United States in recent years (1, 2). These trends underscore the societal relevance and significance of unions, highlighting the need to understand the forces influencing workers’ interest in joining, and support for, unions. Prior research consistently finds that perceptions of the material benefits associated with union membership are positively associated with interest in joining unions, and support for organized labor more generally (3, 4). However, past research leaves open important questions about these relationships, in particular: i) how accurate Americans’ perceptions are and ii) whether these positive associations reflect a causal impact of expected benefits on interest in joining, and support for, unions.

Regarding the first open question, there are good reasons to think that Americans may systematically underestimate material benefits associated with unionization. On the one hand, unions have been targeted by negative campaigns led by employer organizations, as well as conservative politicians and groups, arguing that the materials benefits of unions are nonexistent or even negative (5). Additionally, declining unionization rates, and increasing regional and professional concentration of union membership, mean fewer people have direct, or indirect, contact with union membership, contact that might foster accurate perceptions of union benefits (6). On the other hand, it’s also possible that material benefits of unionization could be overestimated, because unions actively seek to foster positive perceptions of unions and their effects through direct organizing of workers and indirectly through political activity.

Regarding the second open question, it is unknown from prior research whether perceptions of material benefits associated with unionization causally influence support for, and interest in joining, unions. While this association seems plausible, it is possible that the correlation between these two variables is the result of reverse causation, i.e., that people may like unions for largely nonmaterial reasons, and after this come to view them as providing significant material benefits to their members. Yet another possibility is that the correlation is entirely spurious, with other variables—such as political ideology, or exposure to politician or peer influence on these views—producing the correlation.

Here, we explore Americans’ perceptions of the material benefits associated with unionization, comparing these against data drawn from the Bureau of Labor Statistics (BLS) and peer-reviewed research. Additionally, we study whether the previously documented positive correlation between these perceptions and interest in joining unions and support for unions reflects a causal relationship. We conducted a preregistered online survey experiment on a sample representative of the U.S. population for a set of benchmarks for gender, age, region, and education (*n* = 1,430). Participants were first asked to estimate levels of material benefits earned by unionized and nonunionized workers in the United States across six domains: total life-time wages, average annual salary, access to health-insurance benefits, having more than 10 d paid time off, access to retirement benefits, access to dental benefits—as well as the average union dues rate. To not bias responses in either direction, the midpoint for all answer scales was set to the value for nonunionized workers.

Participants were then randomly assigned to a correction condition, where they saw a summary table with their own answers next to correction statistics, with sources explicitly noted (*SI Appendix*), or a control condition, where the table featured only their own answers. Next, respondents answered a number of survey questions, including items tapping their interest in joining unions and general support for unions—our two primary, preregistered outcomes—as well as their willingness to undertake costly action in support of unions (e.g., helping to organize a union in their workplace) and their support for several pro-union policies (e.g., support for repealing state “right to work” laws).

## Results

**Fig. 1.**
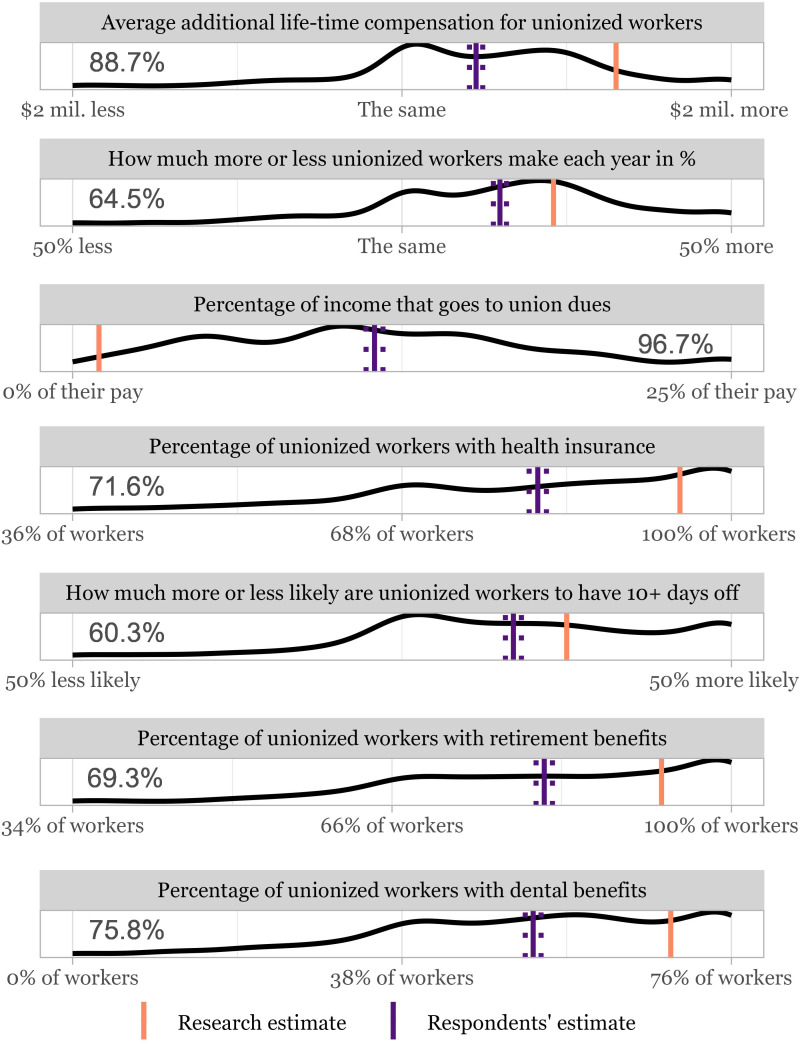
Kernel-density distributions of responses for all material benefits items, as well as the percent of respondents who underestimated union benefits. Dotted lines next to the respondents’ estimate indicate the 95% CI from a *t* test where the H0 is the research estimate. *X*-axis labels are the same as question anchors used in the survey.

Fig. 1 presents the distribution of responses for the seven benefit perception items, as well as the percentage of respondents who underestimated the material benefits of unions on each item. These results show that a large majority of Americans underestimated the material benefits of unions. For example, 88.7% of respondents underestimated the life-time compensation premium associated with union membership, and 96.7% overestimated the average rate of union dues. Respondents were most accurate in estimating the percentage of unionized workers with more than 10 d of paid vacation, though the majority (60.3%) still underestimated the rate of this union benefit.

Next, we analyze correlates of perceived union benefits among respondents in the control condition using a composite index of standardized measures of the perceived levels for all seven response items. Among participants in our study, underestimates of union benefits were more prevalent among younger people (Pearson’s *r* = − 0.21, *P* < 0.001), people who know fewer union members (*r* = 0.18, *P* < 0.001), less educated people (*r* = 0.15, *P* < 0.001), racial minorities (*r* = 0.13, *P* = 0.001), conservatives (*r* = 0.12, *P* = 0.001), and people who were not current union members (*r* = 0.11, *P* = 0.004). Additionally, we find underestimation of union benefits was negatively correlated with support for unions (*r* = −0.31, *P* < 0.001), and interest in joining them (*r* = −0.21, *P* < 0.001), consistent with prior research.

**Fig. 2.**
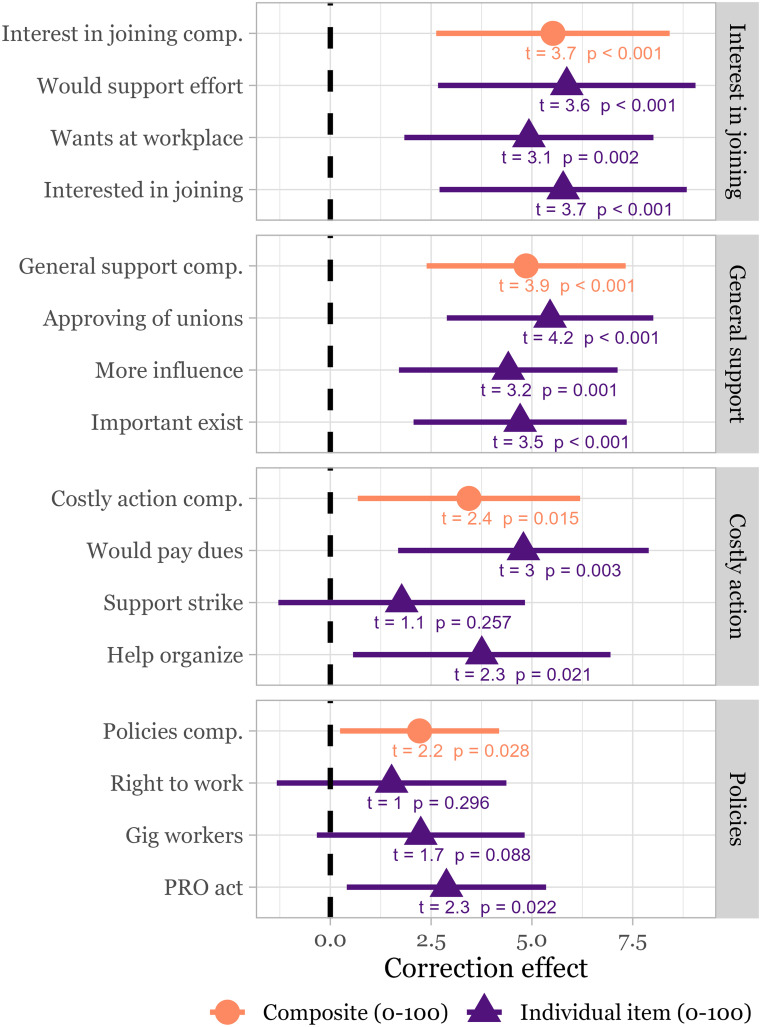
Estimated treatment effects of the correction treatment updating respondents’ perceptions of the material benefits of unions (with controls for age, gender, education, income, race/ethnicity, partisanship, and being a union member). 95% CIs for the unstandardized coefficients are shown.

We next conduct preregistered analyses of the effects of the correction treatment (see *Data, Materials, and Software Availability* for the preanalysis plan), in which treated participants were presented with a table featuring their responses and estimates drawn from prior governmental and academic research. Fig. 2 presents these results. Mean levels for the control group for each of the dependent variables, measured via multiple items on 100-point scales and averaged to form composites, are 62.2 (general support), 47.6 (interest in joining), 49.6 (willingness to take costly action), and 59.6 (support for pro-union policies). Respondents who received the correction treatment reported significantly greater interest in joining unions (β=5.52, *t* = 3.7, *P* < 0.001), general support for unions (β=4.86, *t* = 3.9, *P* < 0.001), willingness to take costly action in support of unions (β=3.43, *t* = 2.4, *P* = 0.015), and greater support for pro-union policies (β=2.21, *t* = 2.2, *P* = 0.028), as compared to the control group. These effects correspond to an 11.6% greater willingness to join unions and 7.8% greater support for unions as compared to the mean levels in the control group. Effects for willingness to take costly action and support for pro-union policies were more modest, though also significant and positive (6.9% and 3.7%, respectively).

## Discussion

Results of our study suggest that misperceptions of the material benefits associated with unionization are widespread in the American mass public: overall, we found 92% of Americans underestimated the material benefits of unions on the composite benefit index. Participants who were shown a brief informational correction reported greater interest in joining a union, general support for unions, willingness to take costly action to support unions, and support for pro-union policies, as compared with participants who did not receive the correction. These results indicate that perceptions of the material benefits associated with unionization are causally linked to these outcomes.

While we used official government reports and peer-reviewed research, many sources are available for estimating the material benefits of unionization, and specific sources used could influence the benefit estimates and the credibility of the estimates to participants. Further, we have focused on perceptions of the material benefits associated with union membership and were careful to present data to study participants in this way. While some of the research we draw upon uses quasi-experimental methods to estimate causal effects of union membership on material benefits (7, 8), such estimates are not available for the full range of benefits and therefore we opted to be conservative in how we presented the benefits of unionization to respondents. It is possible that if we had data on, and asked about, views of causal union membership benefits, our results would have been different, a topic that future work should investigate.

Our results suggest interest in joining unions would be higher if not for significant underestimates of the material benefits associated with unionization. These misperceptions may help explain why interest in joining unions in the United States continues to lag behind peer countries, in addition to other previously identified factors, such as the relatively low importance of class identity in the United States, as well as legal and structural impediments to unionization (9). Future studies should test whether corrections such as the one studied here influence observable behavioral outcomes in real-world contexts.

## Materials and Methods

From February 7 to 14, 2023, we recruited 1,430 participants via Bovitz’s Forthright Panel to the study. We included participants who passed two pretreatment, preregistered attention checks. The sample was representative of the U.S. population on a set of benchmarks for gender, age, region, and education. Quota-matching was assessed after the attention checks. Participants were randomly assigned either to a correction condition, where they were shown their own answers on seven items asking them about the material benefits of unions next to the correct statistics, or a control condition, where participants were only shown their own answers. The correct statistics come from the BLS, who collect data through surveys conducted by the Bureau of Census, and peer-reviewed research. Average union dues were estimated from publicly reported data from major unions. Next, respondents were asked questions about 1) their interest in joining unions, 2) general support for them, 3) their willingness to undertake costly action in support of unions, 4) and support for a number of federal policy proposals favorable to unions. For each outcomes, we asked multiple survey items on 0 to 100 scales and averaged responses to form composites for each outcome (Cronbach’s alpha’s = 0.94, 0.94, 0.88, 0.65, respectively). To verify answer scaling did not bias results, we conducted a survey (*n* = 350) measuring Americans’ misperceptions on the same dimensions, but answers were provided via free-response text boxes. Respondents’ average estimates using this format are very similar to those reported in Fig. 1: $31,809, 12.4%, 11.4%, 76.9%, 29.7%, 76.4%, 66.5%, respectively (*SI Appendix*, section A.11 for additional methodological detail). Research was approved by the Stanford University Institutional Review Board. All subjects provided informed consent.

## Supplementary Material

Appendix 01 (PDF)

## Data Availability

Preregistration, data, and analysis code files have been deposited in Open Science Framework (https://osf.io/c6kp9/) (10). Full text of treatments and survey items are in *SI Appendix*. Statistics on union benefits come from the BLS and peer-reviewed research. Average union dues were estimated from publicly reported data from major unions. Detail on these statistics can be found in *SI Appendix*.

## References

[1] T. A. Kochan , An overview of US workers’ current organizing efforts and collective actions. Work Occup. **50**, 335–350 (2023).

[2] J. McCarthy, U.S. approval of labor unions at highest point since 1965 (Gallup) (2023). https://news.gallup.com/poll/398303/approval-labor-unions-highest-point-1965.aspx. Retrieved 20 October 2023.

[3] T. A. DeCotiis, J. Y. LeLouarn, A predictive study of voting behavior in a representation election using union instrumentality and work perceptions. Org. Behav. Hum. Perform. **27**, 103–118 (1981). 10.1016/0030-5073(81)90041-6 10249822

[4] H. Park, P. P. McHugh, M. M. Bodah, Revisiting general and specific union beliefs: The union-voting intentions of professionals. Ind. Relat. **45**, 270–289 (2006).

[5] K. Bronfenbrenner, No holds barred: The intensification of employer opposition to organizing (2009). https://www.epi.org/publication/bp235/. Accessed 20 October 2023.

[6] A. Hertel-Fernandez, S. Naidu, A. Reich, Schooled by strikes? The effects of large-scale labor unrest on mass attitudes toward the labor movement. Perspect. Polit. **19**, 73–91 (2021).

[7] J. S. Ahlquist, Labor unions, political representation, and economic inequality. Annu. Rev. Polit. Sci. **20**, 409–432 (2017).

[8] T. Van Heuvelen, The right to work and American inequality. Am. Sociol. Rev. **88**, 810–843 (2023).

[9] B. Eidlin, Labor and the Class Idea in the United States and Canada (Cambridge University Press, 2018).

[10] J. Kamphorst, R. Willer, Data from “Correcting Misperceptions of the Material Benefits Associated with Union Membership Increases Americans’ Interest in Joining Unions.” Open Science Framework. https://osf.io/c6kp9/. Deposited 12 April 2024. 10.1073/pnas.2321025121 PMC11087758 38683999

