# Supplementary material for: Correcting misperceptions of the material benefits associated with union membership increases Americans’ interest in joining unions
Source: Proc Natl Acad Sci U S A. 2024 Apr 29;121(19):e2321025121. doi: 10.1073/pnas.2321025121 (PMC11087758; doi:10.1073/pnas.2321025121)
Supplement: Supplementary file 1 — Appendix 01 (PDF) [file pnas.2321025121.sapp.pdf]

A Supporting Information

A.1 Treatment conditions and reproduction files:

The full treatment conditions, data, analysis code, pre-analysis plan, and survey tools can be found on the OSF online repository: [https://osf.io/c6kp9/.](https://osf.io/c6kp9/)

A.2 "True" answers on the benefits items and sources for the answers

Participants were asked their perceptions of material benefits of unions on seven items (life-time wage benefits, yearly salary, union dues, health-insurance benefits, time-off, retirement benefits, and dental benefits). Treated respondents were shown the correct statistic next to their own answer. The wording for the misperception items in the treatment condition is below. Treated respondents saw a message like this for all seven scales on the same page next to their own answer.

- *Additional lifetime income*. The correct figure is that unionized workers make $1.3 million more over the course of their lifetime, on average in the U.S. compared to non-unionized workers. This research was discovered by a team led by researchers from the University of Minnesota. The study was published in a journal from Cornell University in 2022.
  - Source: Parolin & Van Heuvelen (2022). "The Cumulative Advantage of a Unionized Career for Lifetime Earnings." In: ILR Review., URL: https://journals.sagepub.com/doi/10.1177/00197939221129261

- *More income in percentages*. The correct figure is that unionized workers earn 23% higher wages than non-unionized workers, on average in the U.S. We know this based on figures from the U.S Bureau of Labor Statistics, which is a government agency. The statistics were published by the government in 2020.
  - Bureau of Labor Statistics (2020). "Nonunion workers had weekly earnings 81 percent of union members in 2019." URL: https://www.bls.gov/opub/ted/2020/nonunion-workers-had-weekly-earnings-81-percent-of-union-members-in-2019.htm

- *Percentage of income in union dues.* The correct figure is that the typical unionized worker pays 1% of their income in union dues in the U.S. We know this based on public information reported by major American unions.
  - https://www.teamsters665.org/dues/https://anh.com/the-cost-of-unions/#:~:text=The%20average%20annual%20cost%20of,want%20full%2Dtime%20dues%20payers.

- *Healthcare through employer*. The correct figure is that 95% of unionized workers are provided health insurance through their employer in the U.S. This figure was published in a report from the Economic Policy Institute in 2021.
  - Economic Policy Institute (2022). "Unions are not only good for workers, they're good for communities and for democracy." URL: https://www.epi.org/publication/unions-and-well-being/#:~:text=Higher%20incomes%20allow%20workers%20and,%2C%20retirement%2C%20and%20paid%20leaveAlsofromBLS.Originalquestiontextis:Percentofprivateindustryworkerswithaccesstohealthcarebenefits:medicalcare;forbargainingstatusisnonunion

- *Paid time off.* The correct figure is that unionized workers in the private sector are 25% more likely to have 10 or more pair days off than non-unionized workers in the U.S. This figure is also from the Bureau of Labor Statistics, which is a government agency. The statistics were published by the government in 2022.
  - Bureau of Labor Statistics (2022). "National Compensation Survey: Employee Benefits in the United States." URL: https://www.bls.gov/ncs/#tables

- *Access to retirement benefits.* The correct figure is that 93% of unionized workers have access to retirement benefits in the U.S. This is based on figures from the Bureau of Labor Statistics. The statistics were published in a government report in 2022.
  - Bureau of Labor Statistics (2022). "National Compensation Survey: Employee Benefits in the United States." URL: https://www.bls.gov/ncs/#tables

- *Dental benefits.* The correct figure is that in the U.S. 69% of unionized workers have access to dental benefits. This figure comes from the Bureau of Labor Statistics. The statistics were published in a government report in 2022.
  – Bureau of Labor Statistics (2022). "National Compensation Survey: Employee Benefits in the United States." URL: https://www.bls.gov/ncs/#tables

The sources from the Bureau of Labor Statistics for paid time off, retirement benefits, and dental benefits come from the "*National Compensation Survey: Employee Benefits in the United States, March 2021.*" This data was downloaded from the BLS website in August 2022. The file can be found under the "Download entire tables" header at this URL. It can also be found under: https://www.bls.gov/ncs/#tables. The exact sources for each of the seven misperception items are as follows:

- *Additional lifetime income.* Source: Parolin & Van Heuvelen (2022)." The Cumulative Advantage of a Unionized Career for Lifetime Earnings." In: *ILR Review*.
- *More income in percentages.* Source: Bureau of Labor Statistics (2020). "*Nonunion workers had weekly earnings 81 percent of union members in 2019.*" https://www.bls.gov/opub/ted/2020/nonunion-workers-had-weekly-earnings-81-percent-of-union-members-in-2019.htm
- *Union dues.* Source: We know this based on public information reported by major American unions as well as a union-busting firm. We rounded up the most accurate figure that we could find (0.963%). The sources:
  - https://guide.unitworkers.com/union-dues-explained/#:~:text=What%20are%20typical%20union%20dues,%241%20for%20every%20%24100%20earned.report that dues are 1-2%.
  - https://ifpte21.org/join/ reports that dues are 0.963% (click on download membership form).

- https://www.teamsters665.org/dues/reports that dues are about 2.5 times someone's hourly wage every month. Assuming a 22 weekdays in a month and an 8-hour workday, that's 22*8 = 176 hours of working. 2.5/176 = 1.4%.
- Union-busting consultancy firms report similar figures: two hours of pay per month, being 2/176 = 1.1% (e.g.: https://anh.com/the-cost-of-unions/#:~:text=The%20average%20annual%20cost%20of,want%20full%2Dtime%20dues%20payers.)

- *Healthcare through employer* Source: Economic Policy Institute (2022). "*Unions are not only good for workers, they're good for communities and for democracy.*" The Economic Policy Institute used statistics from the Bureau of Labor Statistics. The original wording for the statistics reported by the BLS: Bureau of Labor Statistics (BLS). 2020. "*National Compensation Survey: Employee Benefits in the United States,*" March 2020.
- *Paid time off*. Source: "*National Compensation Survey: Employee Benefits in the United States, March 2021.*" See "Table 34. Paid holidays: Number of days provided, private industry workers, March 2021."
- *Access to retirement benefits.* Source: Bureau of Labor Statistics (2021). "*National Compensation Survey: Employee Benefits in the United States, March 2021.*" See "Table Retirement benefits: Access, participation, and take-up rates, private industry workers, March 2021."
- *Dental benefits*. Source: Bureau of Labor Statistics (2021). "*National Compensation Survey: Employee Benefits in the United States, March 2021.*" See "Table 10. Healthcare benefits: Access, participation, and take-up rates, private industry workers, March 2021— continued."

A.3 Composite Misperception index construction

For each of the seven individual misperceptions items, we create a variable that captures misperceptions on the item. We do so by taking the true value and subtracting a respondent's guess. On these new variables, 0 means having no misperceptions, and positive (negative) values mean having misperceptions where someone thinks unions have fewer (more) benefits than they do. We further combine misperceptions on all seven variables into a single index. We do so by dividing each of the items by their standard deviation, followed by taking the mean of all items. Similar to the individual items, on the index positive (negative) values means having misperceptions, on average, where someone thinks unions have fewer (more) benefits than they actually do—0 means not having any misperceptions.

To show the correlates (using Pearson's R) between this index and several respondent co-variates, we rely on several measures:

1. Age in years.
2. Number of people that someone knows that are in a union.
3. Self-reported education, modeled as a continuous variable with three levels.
4. A binary variable for whether someone is a minority.
5. Whether someone is conservative on a 7-step POLIT ideology score.
6. A binary variable capturing whether people are in a union themselves.

A.4 Participants

We excluded participants who failed either of the two pre-manipulation attention checks. The final sample has 1,430 participants and was 49% male, had a median age of 45, was 65% white, 36% had a college degree or higher, 20% were in a union, and 40% of the respondents considers themselves a Democrat. The final sample was representative on gender, age, region, and education. Quotas were determined after the attention checks.

A.5 Analysis

As pre-registered, we compare respondents in the treatment and control conditions using multivariate regression models. Our estimand of interest is the Average Treatment Effect. We ran the following linear model in R:

Outcome = condition + age + female + income + race + education + partisanship + union member

Where income is a continuous variable and education (in three categories), gender, age (continuous), race, partisanship (REP, IND, DEM), and being a union member are dummy variables. All control variables are measured pre-treatment. We use the standard p <0.05 criteria for a two-tailed test to determine significance.

A.6 Moderation and mediators

While we did not pre-register any heterogeneity analysis, we used Causal Forests models among our pre-registered control variables to check if there are any heterogeneous treatment effects. The measure of importance that we used is a simple weighted sum of how many times feature i was split on at each depth in the forest. The causal forest indicates that age (0.48), income (0.13), and partisanship (0.11) are the most important moderators. However, in a multivariate regression model none of these variables show significant interactions with the treatment at the 95% significance level. We stick to what we pre-registered and do not report any heterogeneous treatment effects.

We additionally included four groups of outcomes with two questions each to capture potential mediators: perceived individual efficacy to improve one's working conditions, respect for the working class, thinking unions help the average American, and thinking unions would help respondents' own material situation. We find a significant effect of the treatment on indexes of these outcomes (0-100) for: respect (β =, 2.99, t = 2.7, p = 0.006), thinking unions help the average American (β = 6.69, t = 4.7,

p < 0.001), and thinking unions help the respondent (β = 6.160, t = 3.9, p < 0.001). We find no significant effects for perceived efficacy (β =, 0.487, t = 0.4, p = 0.682).

A.7 Deviations from the PAP

The PAP can be found on the OSF online depository: https://osf.io/c6kp9/

Thirty people identified as "other gender," so we added this category as separate dummy among the control variables. The results do not change if we include these respondents into the"male" or "female" categories of the gender variable.

Furthermore, in the PAP we explicitly mentioned four hypotheses for clarity. To stay within the word limit, we did not repeat these hypotheses in the paper. We did include the results for the pre-registered tests for each hypothesis in the main text. All hypotheses were supported. These pre-registered hypotheses were:

- **H1**: People will underestimate the benefits of unions.
- **H2:** There will be a correlation between the perceived benefits of unions and supporting unions.
- **H3:** Providing information about the material benefits of unions will lead to an increase in general support for unions.
- **H4:** Providing information about the material benefits of unions will lead to an increase in interest in joining a union.

A.8 Attention checks

Attention checks:

- To help us keep track of who is paying attention, please select "Somewhat disagree" in the options below. [Strongly agree: Strongly disagree, 5-step]
- Please read the following short article. Officials in a midsize town have been working for four years on a plan to produce an event license to cover all of the major events that occur at the town's local stadium, which hosts concerts and home sports games. The application would be submitted each

January and list all events expected to occur at the stadium over the next 12 months. If an unlisted event emerges during the year, lawmakers could hold a special hearing on the event, or accept it without a hearing and add it into the existing license. To assist with this plan, lawmakers filed legislation that would change state licensing laws so that annual event licenses will expire within one year. “This makes a minor change to current law, which provides that all licenses issued shall expire on December 31 of each year,” a lawmaker said. What was the topic of the short article you just read about? [Medical funding, Event licensing, Political polarization, City budgeting, Election monitoring policy, Campaign finance reform]

A.9 Consent form

You are invited to participate in a research study that will ask about your opinions and attitudes. You must be at least 18 years of age to participate. There are no risks associated with this study and your identity will be kept confidential. We cannot and do not guarantee or promise that you will receive any benefits from this study.

Participation

If you decide to participate in this project, please understand your participation is voluntary and you may withdraw your consent or discontinue participation at any time without penalty. The alternative is not to participate. You have the right to refuse to answer particular questions. Your individual privacy will be maintained in all published and written data resulting from the study.

Contact Information

If you have any questions, concerns or complaints about this research, its procedures, risks and benefits, contact the Protocol Director, Robb Willer at willer@stanford.edu. If you wish to contact someone independent of the researchers, you may email the Stanford Institutional Review Board at irb2-manager@lists.stanford.edu.

If you agree to participate in this research, please click to the next screen and complete the questionnaire.

A.10 Exact question wording of the outcome variables and outcome-index construction

Our outcomes are measured on 0-100 scales. As our main outcome variables, we create several indexes that combine different items (as pre-registered). We create these indexes by adding up the individual scales and dividing them by the number of variables. Both the indexes and their constituent variables thus range from 0 to 100. For our main outcomes, we create the following indexes by combining the following items:

- *General support index:*
  - I support labor unions.
  - I would personally like labor unions in the U.S. to have more influence than they have today.
  - It is important that labor unions exist.

- *Personal interests in joining index:*
  - If there was an effort to unionize at my workplace, I would support it.
  - I hope that my workplace becomes unionized.
  - I am interested in joining a labor union

We additionally include three other composite variables as outcomes in the experiment:

- *Costly Action:*
  - If my workplace became unionized and paying full dues was optional, I would choose to pay full dues.
  - If there was an effort to unionize at my workplace, I would help organize it.
  - If there was a strike at my workplace, I would take part.

- *Policy support:*
  - Have you heard of "gig workers" and their efforts to unionize? "Gig workers" are people who work in jobs where it seems like they are self-employed," such as Uber drivers, food delivery drivers, and Amazon delivery drivers. Because some people argue that gig workers are self-employed and don't work for a boss, it is hard for them to unionize in many states. There is a discussion about the need for legislation that allows gig workers to unionize. Do you support or oppose legislation that makes it possible for gig workers to form unions?
  - Have you heard of "right to work laws"? "Right to work laws" prohibit unions from requiring that workers pay dues to the union. Those who oppose getting rid of these laws say that people should be able to choose to pay dues or not. Those who support getting rid of these laws say the laws make unions weaker and that everyone who benefits from the union should pay dues. Do you support getting rid of "right to work laws"?
  - Have you heard of the "Protecting the Right to Organize Act", or "PRO Act"? The PRO Act is a proposed United States law that would expand various labor protections such as the right to organize and the right to join a union. Do you support or oppose the "PRO Act"?

A.11 On measuring misperceptions and scale anchors

The question scales asking about respondents' misperceptions (Figure 1) were formatted in such a way that the mid-point was the value for non-unionized workers (this was not the case for the union dues question where there is no non-unionized worker reference value). For example, consider the following question: "On average, how much do you think <u>unionized workers</u> make in a year, as compared

with <u>non-unionized workers in the U.S.</u>?" The mid-point on the answer scale for this question was "'The same," the anchors at the beginning and end of the scale were "50% less" and "50% more." This way of asking the questions ensured that we did not bias respondents towards indicating that being in a union is better or worse than not being in one, since the mid-point is there being no difference between unionized and non-unionized workers. To additionally prevent bias due to the answer scale format, respondents had to click on the scale before the answer slider-bar appeared (as opposed to having the slider bar default to the midpoint or one of the anchors).

To further ensure that our findings are not driven by our answer scales, we ran an additional survey as a robustness check using the same sample provider and sampling quotas (n = 350), as reported in the Materials and Methods section. In this survey, we reformatted the questions so that responses would be given in free-response format, with participants including whatever value they wanted in a text box. For example, the questions asking about the correct value for unionized workers with a specific benefit (e.g. health insurance) asked for a percent in an open-ended text box. For the questions asking about how much more or less likely a given benefit was for unionized workers, we first asked whether respondents thought unionized workers would get "more." "less," or "the same," and then provided respondents with a follow-up text box where they could specifically enter how much more or less. While we prefer the questions used for the main paper because they make it easier for participants to answer the survey, this robustness check ensures that there are no scaling effects. As the results reported in the Materials and Methods section show, respondents' average estimates using this question format are very similar. The exact question wording for these additional robustness items can be found below:

- *Life-time income benefits of union membership.*
    - Over the course of someone's entire working career, do you think a <u>unionized worker</u> makes **<u>more</u>**, **<u>less,</u>** or **<u>the same</u>** as compared to a <u>non-unionized worker, on average in the U.S.</u>? (Assuming the unionized worker was in a union for their entire working career.)
    - <u>[IF more/less] **How much more**</u> do you think they make? (Please give your response in total dollars)

- [IF more/less] **How much less** do you think they make? (Please give your response in total dollars)

- *How much more yearly compensation in percentages.*
  - On average, do you think unionized workers make **more**, **less**, or **the same** in a year, as compared to non-unionized workers in the U.S.?
  - [IF more/less] On average, **how much more** do you think unionized workers make in a year, as compared to non-unionized workers in the U.S.? (Please give your response in percentages)
  - [IF more/less] On average, **how much less** do you think unionized workers make in a year, as compared to non-unionized workers in the U.S.? (Please give your response in percentages)
- *Percentage of income to Union dues.*
  - What percentage of their income do you think a typical unionized worker pays in union dues in the U.S.?
- *Percentage of unionized workers with health insurance.*
  - In the U.S., 68% of non-unionized workers have access to health insurance through their employer. What percentage of unionized workers do you think have access to health insurance through their employer?
- *How much more likely to have paid time off.*
  - In the U.S., 28% of non-unionized private sector workers have access to 10 or more days of paid time off. Do you think unionized private sector workers are **more** or **less** likely to receive 10 or more paid days off a year?
  - [IF more/less] **How much more likely** do you think unionized private sector workers are to receive 10 or more paid days off a year?
  - [IF more/less] **How much less likely** do you think unionized private sector workers are to receive 10 or more paid days off a year?
- *Percentage of unionized workers with retirement benefits.*

- In the U.S., 66% of non-unionized private sector workers have access to retirement benefits. What percentage of unionized workers do you think have access to retirement benefits?

- *Percentage of unionized workers with dental benefits.*
  - In the U.S., 38% of non-unionized private sector workers have access to dental care benefits. What percentage of unionized workers do you think have access to dental care benefits?
